# Supplementary material for: Blocking mitochondrial pyruvate import in brown adipocytes induces energy wasting via lipid cycling
Source: EMBO Rep. 2020 Dec 4;21(12):e49634. doi: 10.15252/embr.201949634 (PMC7726774; doi:10.15252/embr.201949634)
Supplement: Supplementary file 1 — Expanded View Figures PDF [file EMBR-21-e49634-s001.pdf]

Expanded View Figures

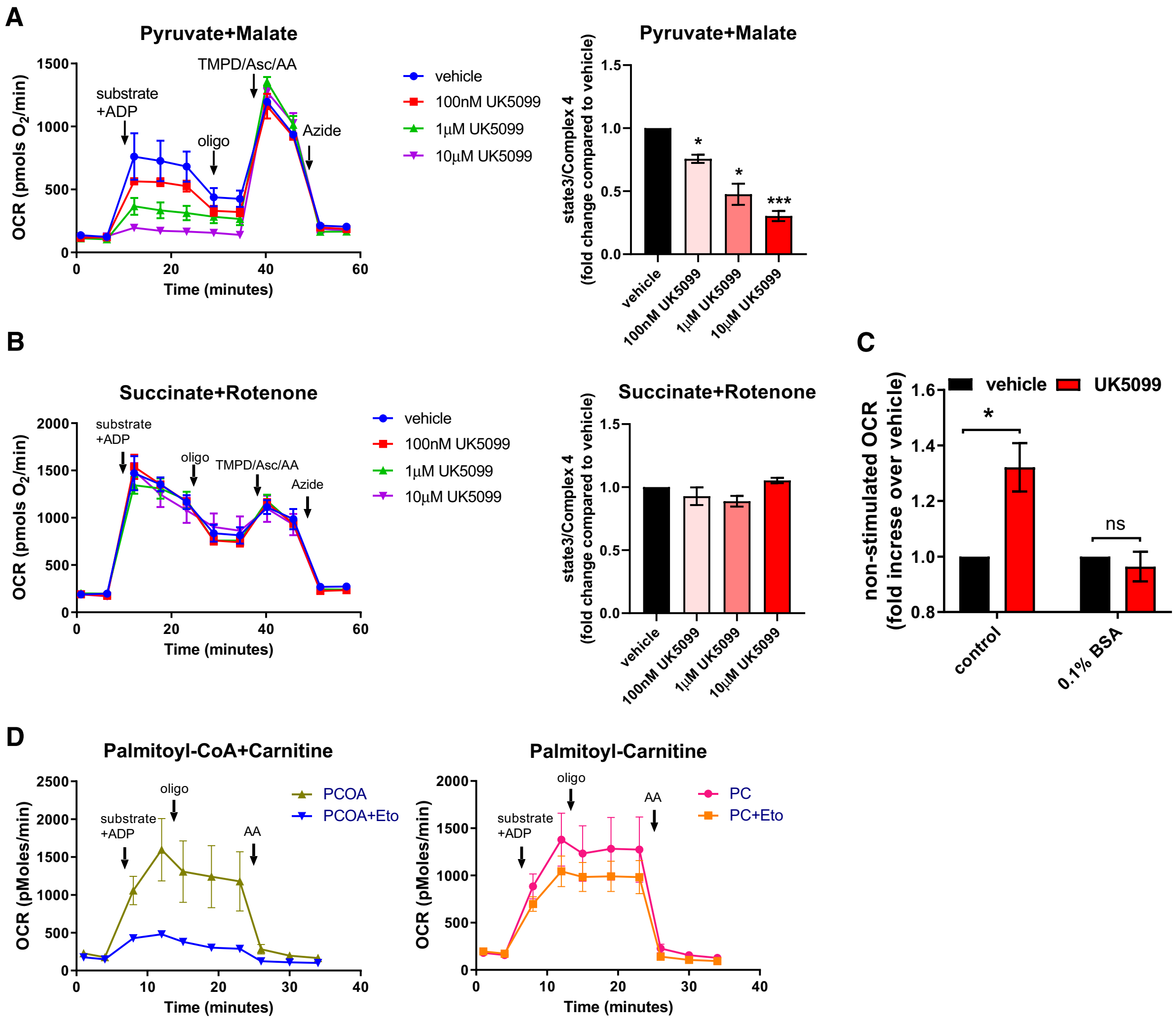

Figure EV1.

 © 2020 The Authors

Figure EV1. Assessment of respiratory rates in permeabilized adipocytes.

A, B Effect of MPC inhibitor UK5099 on respirometry in permeabilized brown adipocytes. (A) OCR of permeabilized cells were measured in presence of 5 mM pyruvate, 0.5 mM malate and 5 mM ADP as substrates and treated with vehicle (DMSO) or indicted concentrations of UK5099. Chart on the left shows representative OCR traces averaging 3 technical replicates. Pyruvate and malate (substrate) + ADP, oligomycin a (Oligo; 4 μM), TMPD (0.5 mM) + ascorbic acid (1 mM) + antimycin A (4 μM) (TMPD/Asc/AA) and sodium azide (50 mM, Azide) were injected where indicated. Bar-graph shows quantification of state 3 OCR normalized to complex 4 activity (measured as maximal TMPD + Ascorbate driven OCR). Data were normalized to vehicle for each individual experiment (n = 4 individual experiments). Note that UK5099 dose-dependently reduced state3/complex 4 OCR. *$P < 0.05$, ***$P < 0.001$ by ANOVA. (B) OCR of permeabilized primary brown adipocytes were measured in presence of 5 mM succinate, 2 μM rotenone and 5 mM ADP as substrates and treated with vehicle (DMSO) or indicted concentrations of UK5099. Chart on the left shows representative OCR traces averaging 3 technical replicates. Succinate and rotenone (substrate) + ADP, oligomycin a (Oligo; 4 μM), TMPD (0.5 mM) + ascorbic acid (1 mM) + antimycin A (4 μM) (TMPD/Asc/AA) and sodium azide (50 mM, Azide) were injected where indicated. Bar-graph shows quantification of state 3 OCR normalized to complex 4 activity (measured as maximal TMPD + Ascorbate driven OCR). Data were normalized to vehicle for each individual experiment (n = 3 individual experiments).

C Brown adipocytes were pre-treated with vehicle (DMSO) or 100 nM UK5099 for 2 h prior to OCR measurements. OCR were measured in Seahorse base media with glucose and glutamine (control) or supplemented with 0.1% fatty acid-free bovine serum albumin (BSA) where indicated. Quantification of non-stimulated OCR of brown adipocytes from n = 3–7 individual experiments. Data were normalized to vehicle for each individual experiment. ns $P > 0.05$, *$P < 0.05$ compared to vehicle by Student's t-test.

D Effect of CPT1 inhibitor etomoxir on respirometry in permeabilized brown adipocytes OCR of permeabilized cells were measured in the presence of 0.1 mM palmitoyl-CoA + 0.5 mM carnitine (PCOA), a substrate that is dependent on CPT1, or in the presence of 0.1 mM palmitoyl-carnitine (PC) a substrate that does not require CPT1 activity. Substrate + ADP, oligomycin a (Oligo; 4 μM), and antimycin A (4 μM) were injected where indicated. Representative OCR traces averaging 3 technical replicates. Note that etomoxir (Eto) only inhibits OCR fueled by palmitoyl-CoA, a substrate that is dependent on CPT1 activity.

Data information: All data are presented as mean ± SEM.

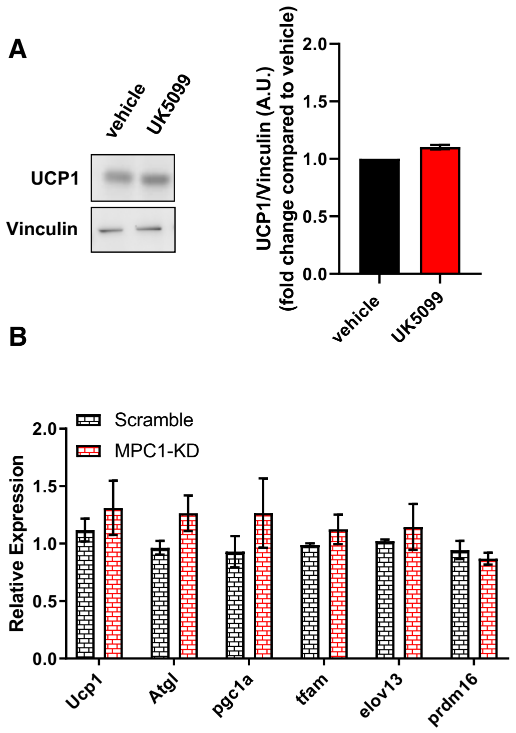

Figure EV2. Pharmacological or genetic blockage of the MPC does not affect brown adipocyte differentiation.

A Primary brown adipocytes were treated with 100 nM UK5099 or vehicle (DMSO) for 4 h. Data show representative Western blot for UCP1 and Vinculin, and quantification of UCP1 normalized to Vinculin from n = 3 individual experiments.

B mRNA levels of UCP1, Atgl, Pgc1α, Tfam, Elov13, and Prdm16 brown adipocytes transfected with scramble RNA (Scramble) or MPC1 siRNA (MPC1-KD) from n = 3 individual experiments. mRNA levels were normalized to 36B4.

Data information: All data are presented as mean ± SEM.

© 2020 The Authors

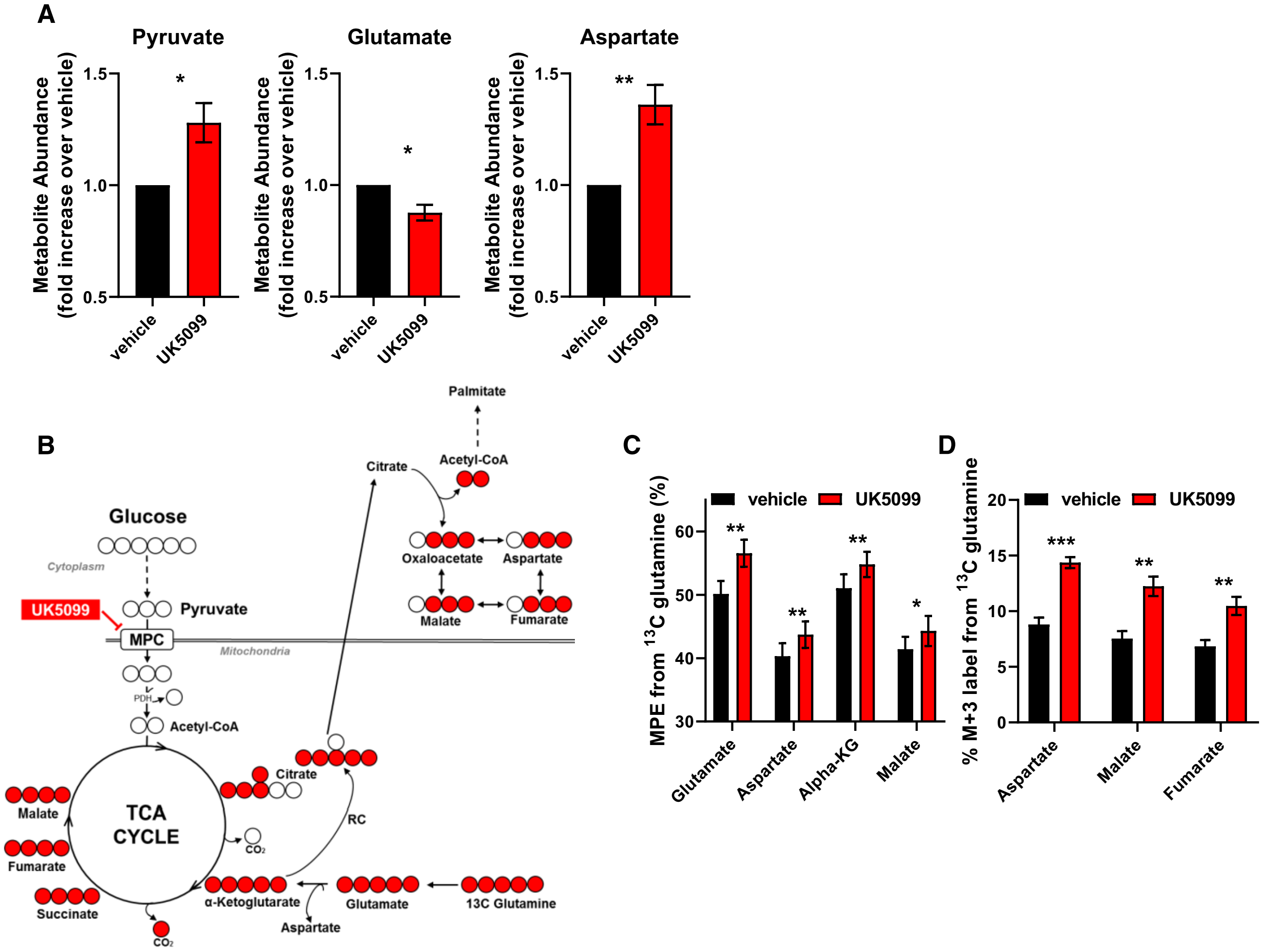

Figure EV3. Effects of UK5099 treatment on cellular metabolites.

A Effect of MPC inhibition on polar metabolite abundance. Primary brown adipocytes were treated for 24 h with vehicle (DMSO) or 10 μM UK5099. Data show total metabolite abundance measured by GC-MS from $n = 6$ individual experiments. Data were normalized to vehicle for each individual experiment. $^*P < 0.05$, $^{**}P < 0.01$ compared to vehicle by Student's *t*-test.

B, C Effect of MPC inhibition on glutamine catabolism and contribution to TCA cycle metabolites. (B) Schematic representation of metabolite tracing using [U-$^{13}C_5$] glutamine. (C) [U-$^{13}C_5$] glutamine tracing in primary brown adipocytes treated with vehicle (DMSO), or 10 μM UK5099. Data show mole percent enrichment (MPE) of isotope-labeled substrate into respective metabolite from $n = 4$ individual experiments. $^*P < 0.05$, $^{**}P < 0.01$ compared to vehicle by Student's *t*-test.

D Quantification of percent M + 3 label from [U-$^{13}C_5$] glutamine into respective metabolite from experiment described in (C) ($n = 4$ individual experiments). $^*P < 0.05$, $^{**}P < 0.01$, $^{***}P < 0.001$ compared to vehicle by Student's *t*-test.

Data information: All data are presented as mean ± SEM.

 © 2020 The Authors

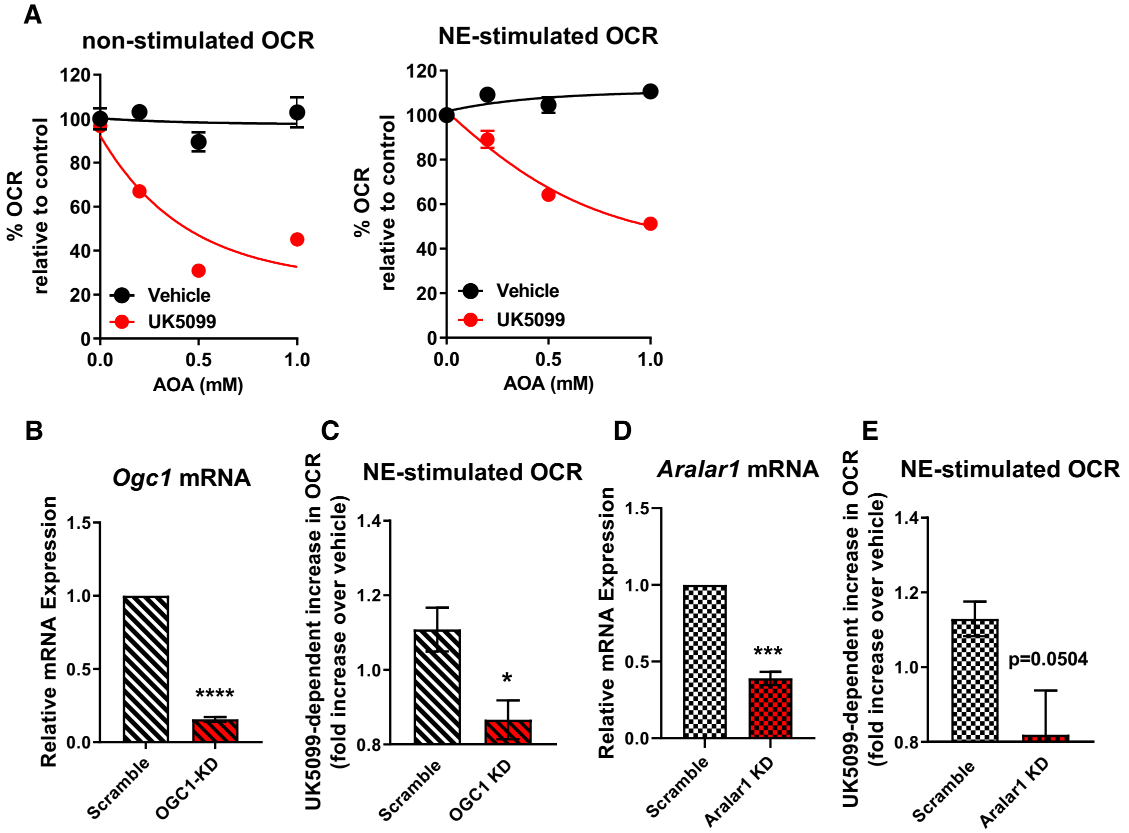

Figure EV4. The malate-aspartate shuttle is required for increased Norepinephrine-stimulated energy expenditure induced by MPC inhibition.

A Brown adipocytes were pre-treated with vehicle (DMSO), 50 nM UK5099, aminooxyacetic acid (AOA) at various concentrations, or a combination. Oxygen consumption rates (OCR) were measured in respirometry media supplemented with 3 mM glucose and 3 mM glutamine in the presence of the compounds. Data show non-stimulated and norepinephrine-stimulated OCR (n = 3 individual experiments). Note that AOA has no effect in vehicle-treated cells, but reduced OCR when cells are treated with UK5099.

B mRNA levels of OGC1 in brown adipocytes transduced with adenovirus carrying either scramble RNA (Scramble) or OGC1 shRNA (OGC1 KD). Data were normalized to vehicle for each individual experiment (n = 3 individual experiments). ****$P < 0.0001$ compared to Scramble by Student's t-test.

C Quantification of norepinephrine-stimulated OCR after vehicle or UK5099 treatment in scramble RNA or OGC1 shRNA transduced cells. Data were normalized to vehicle for each individual experiment (n = 5 individual experiments). *$P < 0.05$ compared to vehicle by Student's t-test.

D mRNA levels of Aralar1 in brown adipocytes transfected scramble RNA (Scramble) or Aralar1 siRNA (Aralar1 KD). Data were normalized to vehicle for each individual experiment (n = 4 individual experiments). ***$P < 0.001$ compared to Scramble by Student's t-test.

E Quantification of NE-stimulated OCR after vehicle or UK5099 treatment in scramble RNA or Aralar1 siRNA-transfected cells. Data were normalized to vehicle for each individual experiment (n = 4 individual experiments). $P = 0.0504$ compared to vehicle by Student's t-test.

Data information: All data are presented as mean ± SEM.

© 2020 The Authors

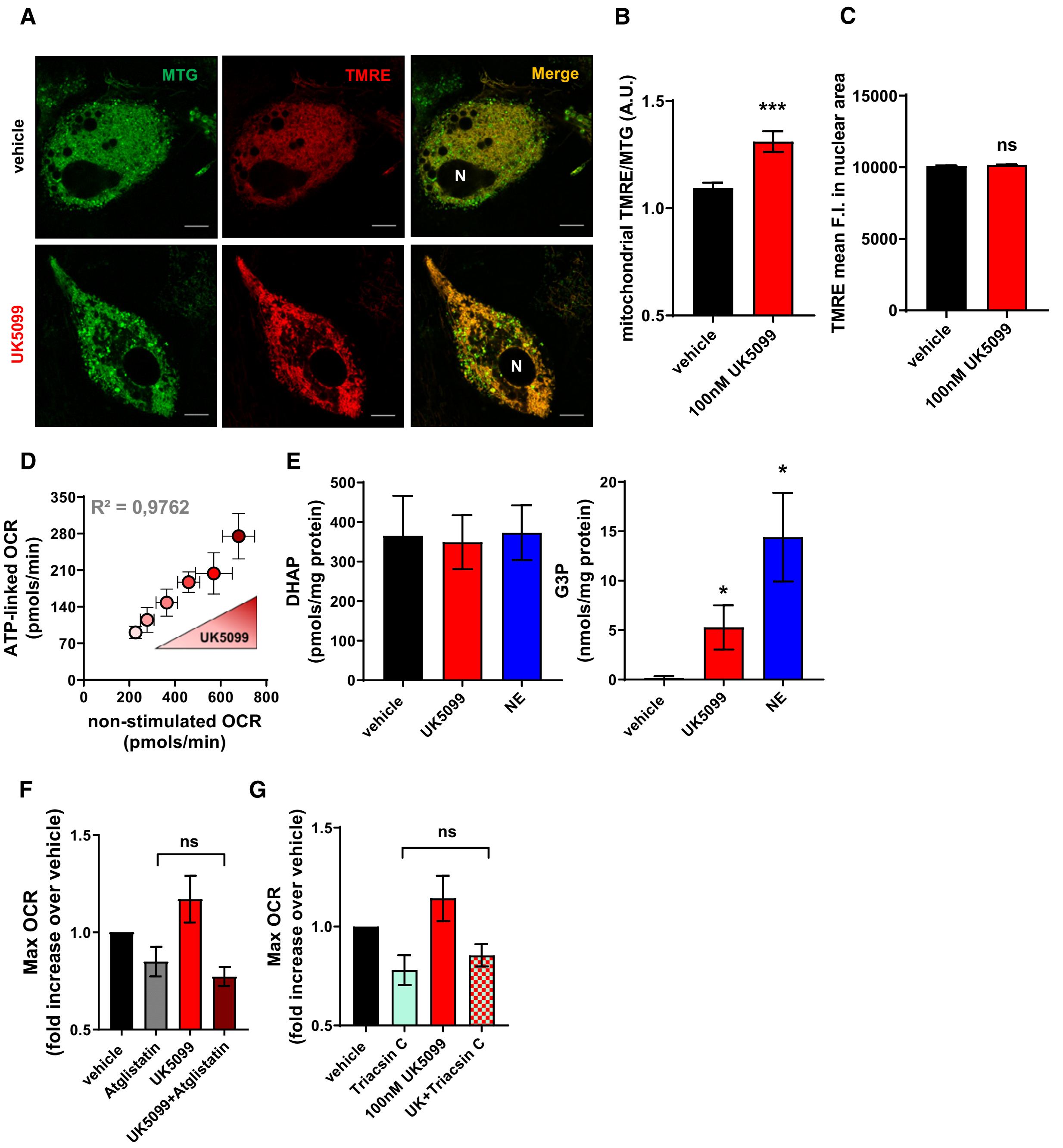

Figure EV5.

 © 2020 The Authors

Figure EV5. Effects of UK5099 on ATP demand and lipid cycling.

A Live-cell super-resolution confocal imaging of primary brown adipocytes. Cells were stained with mitotracker green (MTG, green) and membrane potential sensitive dye TMRE (red). Cells were treated with vehicle (DMSO) or 100 nM UK5099 for 2 h. N, nucleus. Scale bar = 10 μm.

B Quantification of mitochondrial TMRE fluorescence intensity (F.I.) normalized to mitochondrial MTG F.I. from images shown in (A). Data represent 22-27 cells from 3 individual experiments. ***$P < 0.0001$ by Student's *t*-test.

C Quantification of TMRE mean F.I. in nuclear area (N) from images shown in (A). Data represent 15 cells from 3 individual experiments. ns $P > 0.05$ by Student's *t*-test.

D Effect of increasing UK5099 concentrations (50 nM, 100 nM, 1 μM, 5 μM, 10 μM) on brown adipocytes respiratory rates. Note that the dose-dependent increase in basal OCR following UK5099 treatment correlates with an increase in ATP-linked OCR ($n = 3$ individual experiments).

E Quantification of G3P and DHAP in brown adipocytes treated with vehicle, 100 nM UK5099 or 1 μM norepinephrine (NE) for 24 h ($n = 3$ individual experiments). *$P < 0.05$ compared to vehicle by ANOVA.

F Brown adipocytes were treated with either vehicle (DMSO), 100 nM UK5099, 40 μM Atglistatin or Atglistatin in combination with UK5099. Maximal respiratory rates (Max OCR) were calculated after uncoupling by using the proton ionophore FCCP. Data were normalized to vehicle for each individual experiment ($n = 3$ individual experiments). ns $P > 0.05$ by ANOVA.

G Brown adipocytes were treated with either vehicle (DMSO), 100 nM UK5099, 5 μM Triacsin C, or Triacsin C in combination with UK5099. Maximal respiratory rates (Max OCR) were calculated after uncoupling by using the proton ionophore FCCP. Data were normalized to vehicle for each individual experiment ($n = 4$ individual experiments). ns $P > 0.05$ by ANOVA.

Data information: All data are presented as mean ± SEM.

© 2020 The Authors
